# Assessing Staff Well-Being in Child and Adolescent Mental Health Services: A Multi-Disciplinary Analysis to Inform Tailored Interventions

**DOI:** 10.1192/bjo.2026.11267

**Published:** 2026-06-30

**Authors:** Sharique Ahmad, Dush Mahadevan

**Affiliations:** 1 Lancashire and South Cumbria NHS Foundation Trust, Preston, United Kingdom; 2 Lancashire and South Cumbria NHS Foundation Trust, Burnley, United Kingdom

## Abstract

**Aims:**

Child and Adolescent Mental Health Services (CAMHS) are navigating a landscape of significant growth in service demand and increasing clinical complexity. To ensure long-term workforce sustainability and maintain the highest standards of patient care, it is essential to understand the various factors influencing staff well-being. This project, undertaken as part of the Royal College of Psychiatrists Leadership Fellowship, sought to evaluate the current well-being landscape across a multi-disciplinary CAMHS workforce. The goal was to identify existing protective factors and pinpoint areas where additional systemic support could be optimised to foster a resilient and stable work environment.

**Methods:**

A baseline assessment was conducted involving 65 multi-disciplinary staff members, including practitioners, psychologists, psychiatrists, and administrators. Using the Copenhagen Burnout Inventory, the project measured three domains: Personal, Work-related, and Client-related well-being. Qualitative feedback was also gathered, identifying primary stressors and the visibility and perception of existing organisational support, providing a data-driven foundation for future Plan–Do–Study–Actcycles, ensuring that subsequent interventions are tailored to the service's needs.

**Results:**

Preliminary findings as of February 2026 indicate a large variation in response rates across teams. While staff remain committed to their roles, notable pressures exist regarding personal (Mean=65.4) and work-related (Mean=55.0) well-being. Significantly, client-related burnout was lower (Mean=40.8), suggesting that direct clinical work remains relatively rewarding. Data identified workload, case complexity, and administrative processes as primary areas for attention.

Qualitative themes highlighted interpersonal support as a significant strength; peer and line-manager relationships were the highest-rated positive influences on resilience. The assessment identified an opportunity to improve visibility of Trust-wide initiatives, as nearly half of respondents were either unaware of available offerings or felt they lacked CAMHS-specific tailoring. This has led to a proposal for creative arts therapies as a tailored resource; these interventions have shown promise in other settings. Feasibility, training, and impact assessment for these are currently under discussion.

**Conclusion:**

CAMHS staff demonstrate high dedication despite systemic pressures. While the strong culture of peer support provides a vital foundation, workforce stability requires both increased resource accessibility and the development of newer, tailored support models. These findings will be shared with the Children and Young People’s Mental Health Board for collaborative action planning. Further breakdown of responses that are team and role-specific will help address locational and job-specific burdens, fostering closer engagement between the Trust and staff to mitigate attrition and ensure a sustainable model of care for the future.

